# Computed Tomography and Coronary Plaque Analysis

**DOI:** 10.3390/tomography11080085

**Published:** 2025-07-30

**Authors:** Hashim Alhammouri, Ramzi Ibrahim, Rahmeh Alasmar, Mahmoud Abdelnabi, Eiad Habib, Mohamed Allam, Hoang Nhat Pham, Hossam Elbenawi, Juan Farina, Balaji Tamarappoo, Clinton Jokerst, Kwan Lee, Chadi Ayoub, Reza Arsanjani

**Affiliations:** 1 Faculty of Medicine, University of Jordan, Amman 11118, Jordan; has0191738@ju.edu.jo (H.A.); rhm2210091@ju.edu.jo (R.A.); 2 Department of Cardiovascular Medicine, Mayo Clinic, 13400 East Shea Boulevard Scottsdale, Phoenix, AZ 85259, USA; abdelnabi.mahmoud@mayo.edu (M.A.); allam.mohamed@mayo.edu (M.A.); farina.juanmaria@mayo.edu (J.F.); tamarappoo.balaji@mayo.edu (B.T.); lee.kwan@mayo.edu (K.L.); ayoub.chadi@mayo.edu (C.A.); arsanjani.reza@mayo.edu (R.A.); 3 Department of Medicine, University of Arizona, Tucson, AZ 85724, USA; npham917@arizona.edu; 4 Department of Cardiovascular Medicine, Mayo Clinic, Rochester, MN 55905, USA; elbenawi.hossam@mayo.edu; 5 Department of Radiology, Mayo Clinic, Phoenix, AZ 85054, USA

**Keywords:** computed tomography, coronary artery disease, plaque analysis, risk stratification

## Abstract

Advances in plaque imaging have transformed cardiovascular diagnostics through detailed characterization of atherosclerotic plaques beyond traditional stenosis assessment. This review outlines the clinical applications of varying modalities, including dual-layer spectral CT, photon-counting CT, dual-energy CT, and CT-derived fractional flow reserve (CT-FFR). These technologies offer improved spatial resolution, tissue differentiation, and functional assessment of coronary lesions. Additionally, artificial intelligence has emerged as a powerful tool to automate plaque detection, quantify burden, and refine risk prediction. Collectively, these innovations provide a more comprehensive approach to coronary artery disease evaluation and support personalized management strategies.

## 1. Introduction

Plaque imaging is a cornerstone in cardiovascular diagnostics, particularly for understanding and managing atherosclerosis, a leading cause of morbidity and mortality worldwide [1]. Advances in imaging modalities have enhanced the identification of vulnerable plaques that are prone to rupture, a precursor to myocardial infarction [2,3]. These vulnerable plaques, characterized by their thin fibrous caps and lipid-rich cores, pose a significant challenge in early detection and accurate risk assessment [2]. The development of non-invasive techniques, like coronary computed tomography angiography, and innovative modalities, like photon-counting CT, has impacted our approach to cardiovascular risk stratification, allowing for a greater understanding of the high-risk features and morphology seen in plaques (Table 1) [4].

### 1.1. Types of CT Imaging Modalities

#### 1.1.1. Dual-Layer Spectral CT Angiography (DL-SCTA)

DL-SCTA has enabled a detailed differentiation between calcified, non-calcified, and mixed plaques [4]. Calcified plaques consist primarily of dense calcium deposits, indicative of advanced atherosclerosis, whereas non-calcified plaques are characterized by softer components, such as lipids or fibrous tissue, with mixed plaques exhibiting a combination of these features that pose unique challenges [4,16]. Utilizing spectral imaging, DL-SCTA assesses the attenuation values of different tissue components at varying energy levels, allowing for the identification of lipid-rich plaques with low-attenuation regions (<30 HU), which are highly vulnerable to rupture and strongly linked to acute coronary syndromes [4,16]. Studies have shown that DL-SCTA improves risk stratification by identifying high-risk features, such as thin fibrous caps, large lipid cores, and intraplaque hemorrhage, subsequently allowing for clinicians to prioritize interventions. For example, a recent study evaluated 35 coronary plaques using DL-SCTA and reported that high-risk plaques exhibited significantly lower delayed iodine enhancement compared to low-risk plaques (1.0  ±  1.5 mg/mL vs. 2.2  ±  1.1 mg/mL, *p*  =  0.021), with a sensitivity of 77% and a specificity of 56% for identifying high-risk plaques [4].

Calcified nodules, varying in size and density, may be characterized by DL-SCTA’s high spatial resolution [4]. Moreover, DL-SCTA enhances the detection of plaque hemorrhage and intraplaque neovascularization, features that signify plaque vulnerability and progression, by leveraging its sensitivity to iodine content [16]. For instance, spectral data analysis has demonstrated that patients with elevated lipoprotein(a) levels have significantly higher plaque attenuation on CT images at 140 keV (36.07 HU vs. 21.32 HU; *p*  =  0.05), supporting a correlation between lipoprotein(a) levels and plaque vulnerability [17]. This imaging modality also allows for the measurement of plaque burden and arterial remodeling, a marker of high-risk plaques where the vessel wall expands outward to compensate for plaque growth [4,16].

Emerging evidence highlights DL-SCTA’s ability to quantify cholesterol crystals and microcalcifications, components linked to increased biomechanical stress and rupture risk, while spectral attenuation curves refine tissue characterization by providing insights into the plaque microenvironment [16]. Combined with advanced motion correction algorithms, DL-SCTA can obtain high-quality imaging in patients with elevated heart rates or arrhythmias, improving diagnostic accuracy and risk stratification and potentially informing personalized treatment strategies for coronary artery disease [4,16].

#### 1.1.2. Photon-Counting CT (PCCT)

PCCT represents an advancement in cardiovascular imaging by measuring individual photon interactions [18]. This innovation improves spatial resolution and reduces image noise, addressing key limitations of traditional CT systems [5,18]. One of its most notable clinical applications is the detection of vulnerable plaques, particularly in high-risk populations, such as patients with diabetes or metabolic syndrome [5]. A study evaluating the performance of PCCT in 300 patients with suspected coronary artery disease, focusing on its ability to differentiate between lipid-rich and calcified plaques, found that PCCT identified lipid-rich plaques with a sensitivity of 94% and specificity of 89%, significantly outperforming conventional CT techniques [5]. Additionally, the study demonstrated its capability to detect thin-cap fibroatheromas, a hallmark of high-risk plaques, with enhanced clarity due to its superior spatial resolution and ability to suppress background noise [5]. PCCT can also accurately detect microcalcifications, often undetectable with traditional imaging, which are emerging as early markers of atherosclerotic disease progression. Through their detection, PCCT provides critical insights into the earliest stages of plaque development, potentially guiding early interventions [19].

Moreover, PCCT provides enhanced visualization of coronary stents, with reduced blooming artifacts that commonly affect conventional CT imaging. A study comparing high-resolution PCCT (HR-PCCT) to conventional CT systems highlighted that HR-PCCT measurements of nonstented and stented plaques were significantly more accurate (nonstented: 4.4% ± 1.1, stented: −9.4% ± 4.6) compared to energy-integrating detector CT (EID-CT) (nonstented: 15.5% ± 4.0, stented: −19.5% ± 5.8) (*p* < 0.001). HR-PCCT also exhibited less change in percent stenosis due to stent artifacts (−5.5%) compared to EID-CT (190.5%) and PCCT (1313%). Observers rated HR-PCCT images as having higher plaque conspicuity and as being least affected by stent artifacts, with a high level of agreement (interclass correlation coefficient = 0.85) [20].

It was demonstrated that ultra-high-resolution PCCT achieved a sensitivity of 96% and a specificity of 84% for detecting coronary artery disease, with an area under the receiver operating characteristic curve (AUC) of 0.93 [6]. Another study on coronary stent patency reported a sensitivity of 100%, specificity of 92.3%, and a negative predictive value of 100%, underscoring its reliability in non-invasive stent evaluation compared to invasive coronary angiography [7].

**Figure 1:**
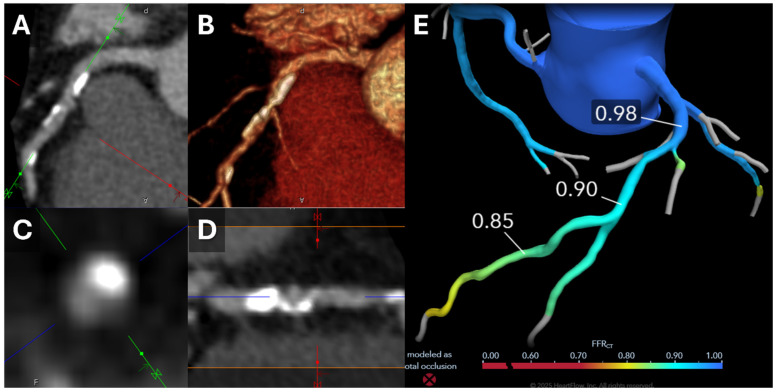
Use of FFRct. 54-year-old man presented to the ED with atypical chest pain; a contrast-enhanced coronary artery CTA was ordered to “rule out coronary artery disease”. Curved centerline 2D (**A**) and 3D (**B**) reconstructions through the proximal and mid-LAD from the CCTA demonstrate partially calcified atherosclerotic plaque. Short-axis (**C**) and straight center line (**D**) reconstructions through the mid-LAD demonstrate a focus of moderate (50–69%) stenosis. The study was sent for FFRct to determine whether the stenosis is hemodynamically significant. FFRct results (**E**) demonstrate a drop in FFRct values from 0.98 to 0.90 across the lesion, indicating a low probability that the stenosis is hemodynamically significant. The patient, based on these results, was medically managed rather than revascularized. The main advantage of FFRct is the ability to predict whether more borderline lesions in the 40–90% range are hemodynamically significant or not. Abbreviations: CCTA = coronary computed tomography angiography, FFRct = fractional flow reserve derived from computed tomography, LAD = left anterior descending artery.

In addition to its diagnostic accuracy, PCCT has shown promise in longitudinal studies aimed at monitoring disease progression in patients undergoing therapeutic interventions. An example of using PCCT to follow a patient with spontaneous coronary artery dissection is illustrated in Figure 1. A recent prospective study followed 120 patients undergoing intensive lipid-lowering therapy over 18 months [21]. PCCT was used to assess changes in coronary plaque composition, revealing a significant reduction in lipid burden and an increase in calcified plaque volume, changes consistent with plaque stabilization. These findings correlated with a reduction in inflammatory biomarkers, such as high-sensitivity C-reactive protein (hs-CRP), reinforcing the potential of PCCT as a tool for evaluating treatment efficacy and tracking disease progression [21].

#### 1.1.3. Dual-Energy CT (DECT)

DECT allows for advanced plaque characterization by utilizing X-rays at two distinct energy levels—typically low and high energy, such as 80 and 140 keV [19,22]. This technique enables simultaneous acquisition of datasets, improving tissue differentiation based on energy-dependent attenuation properties [19]. For instance, lipid-rich plaques demonstrate lower attenuation at lower energy levels, while fibrotic and calcified plaques exhibit distinct attenuation patterns that can be accurately distinguished through material decomposition techniques. These capabilities provide critical insights into plaque composition, identifying markers associated with rupture risk [23,24,25].

Clinical studies have validated DECT’s utility in identifying high-risk plaque features, such as necrotic cores and spotty calcifications [5,24,26,27,28]. In a multicenter study involving 500 patients with suspected coronary artery disease, DECT demonstrated a robust correlation between plaque composition and the incidence of myocardial infarction [5]. The study revealed that patients with necrotic core-dominant plaques had a three-fold higher risk of myocardial infarction over a 12-month follow-up period compared to those with predominantly fibrotic plaques [5].

DECT leverages differences in photoelectric energies and K-edges of materials to distinguish between various plaque components, with iodine and calcium showing higher K-edges (33.2 keV and 4.0 keV, respectively). This differentiation capability improves the assessment of mixed and calcified plaques [22]. Moreover, a simulation study demonstrated that DECT could quantify the chemical composition of coronary artery plaques with high accuracy [8]. For non-calcified plaques, the root mean squared error (RMSE) was 0.7% for water, 1.5% for lipid, and 0.3% for protein contents. For calcified plaques, the RMSEs for 5 mm lesions were 5.6% (water), 5.7% (lipid), 0.2% (protein), and 3.1% (calcium), reflecting the precision of DECT in material differentiation [8].

A retrospective study assessed DECT’s ability to predict imminent ipsilateral ischemic strokes within 30 days. Significant associations were found between subsequent strokes and plaque characteristics, including plaque thickness, with an odds ratio (OR) of 1.59 (95% confidence interval [CI], 1.12–2.24; *p* = 0.009); degree of stenosis (OR = 1.05; 95% CI, 1.02–1.09; *p* = 0.002); plaque ulceration (OR = 20.00; 95% CI, 3.42–116.80; *p* = 0.001); and intraplaque hemorrhage (OR = 7.22; 95% CI, 1.45–35.93; *p* = 0.016). These findings highlight DECT’s potential in identifying high-risk plaques prone to causing acute ischemic events [9].

#### 1.1.4. CT-Derived Fractional Flow Reserve (CT-FFR)

CT-FFR combines anatomical and hemodynamic assessments, allowing for clinicians to evaluate the functional significance of coronary stenoses [29]. By utilizing computational fluid dynamics or machine learning algorithms applied to coronary CT angiography (CCTA), CT-FFR calculates the fractional flow reserve at each point within the coronary vasculature, providing a non-invasive alternative to traditional, wire-based FFR measurements [30]. This approach significantly reduces procedural risks and costs while maintaining high diagnostic accuracy [31].

CT-FFR has shown incremental prognostic value in predicting long-term cardiovascular outcomes. Studies have demonstrated that a CT-FFR value of ≤ 0.80 is strongly associated with adverse outcomes, including major adverse cardiac events (MACE), with hazard ratios as high as 5.05 (95% CI: 3.64–7.01; *p* < 0.001) [26]. For instance, in diabetic cohorts, a CT-FFR ≤ 0.80 independently predicted MACE, with a hazard ratio of 4.534 (*p* < 0.001) [32]. Furthermore, patients with obstructive coronary artery disease and a CT-FFR ≤ 0.80 had significantly higher rates of events like myocardial infarction and revascularization compared to those with non-obstructive CAD or higher CT-FFR values [33,34,35,36,37]. At a 3-year follow-up, the incidence of stable angina and MACE remained substantially elevated in the ischemic group (CT-FFR ≤ 0.80) compared to the non-ischemic group [38].

**Figure 2:**
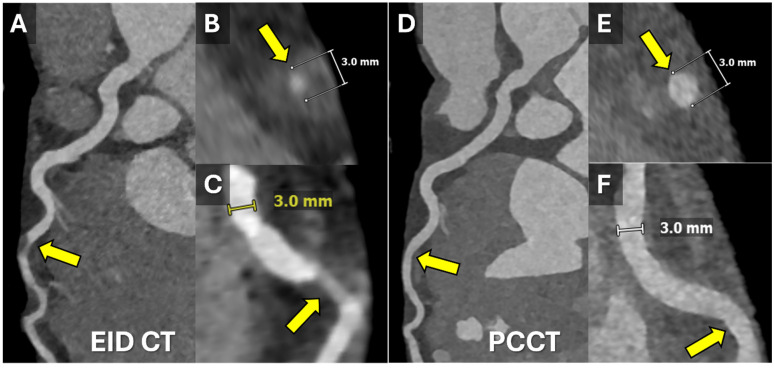
Use of Energy-Integrating and Photon-Counting Detectors. CCTA images from a 38-year-old woman with SCAD affecting the mid-LAD. The SCAD was first detected on a CCTA performed on a CT scanner with a standard energy-integrating detector (EID, (**A**–**C**)). The segment affected is indicated with arrows. A 1-month follow-up CT performed on a CT scanner with photon-counting detectors (PCCT, (**D**–**F**)) demonstrated spontaneous resolution of the SCAD (arrows). The minimum slice thickness on the EID CT is 0.6 mm compared to 0.2 mm on the PCCT. Note the improved spatial resolution (decreased blur) of PCCT, especially on the zoomed-in images ((**B**,**C**) vs. (**E**,**F**)). A 3 mm measurement on images is for purposes of scale. This improved spatial resolution makes it easier to detect subtle coronary lesions and significantly reduces blooming artifacts from coronary stents and heavily calcified atherosclerotic plaques. Abbreviations: CCTA = coronary computed tomography angiography, LAD = left anterior descending artery, SCAD = spontaneous coronary artery dissection.

Incorporating CT-FFR into clinical workflows enhances the specificity of CCTA by reducing unnecessary invasive coronary angiography and downstream costs [10], as shown in Figure 2. Patients with a CT-FFR ≥ 0.80 are less likely to require revascularization and have a trend toward better outcomes [10]. This dual evaluation of anatomical and functional aspects aids in clinical decision-making and procedural planning, ensuring better downstream management, optimization of resource use, and risk stratification [10,39,40,41,42]. It is especially valuable in high-risk populations, including individuals with diabetes, metabolic syndrome, or familial hypercholesterolemia, where it improves risk stratification and early detection of vulnerable plaques characterized by low attenuation, spotty calcifications, and positive remodeling [43]. Emerging evidence highlights the utility of CT-FFR in guiding therapeutic decisions and monitoring treatment efficacy. For example, it has been instrumental in assessing the benefits of novel therapies like PCSK9 inhibitors targeting lipid-rich plaques [43,44,45].

Despite its advantages, CT-FFR has limitations. Extensive coronary calcification and metallic stents can reduce accuracy due to artifacts that hinder lumen segmentation. In scenarios with high Agatston scores (>400), calcium blooming artifacts may compromise the identification of vessel lumens, affecting the diagnostic performance [11,12]. Similarly, metallic stents can cause blooming artifacts that lead to artifactual lumen narrowing, challenging the accuracy of CT-FFR in post-PCI cases [12]. In-stent restenosis detection with CT-FFR, although possible, requires manual editing of the luminal border, which is time-intensive and not always practical. For example, studies show that CT-FFR achieves 85.7% accuracy in diagnosing ISR, but the manual editing process takes an average of 33.5 min per case, which is a limiting factor. In contrast, CTCA alone, particularly with newer scanners, can achieve a higher accuracy of 94.1%, making it a more practical option in certain scenarios [13,14,15]. Additionally, patients with recent myocardial infarction (within 1 month) may exhibit reduced coronary flow dynamics, leading to inaccurate CT-FFR values. This is because the reduction in activity during the post-STEMI recovery phase can lead to decreased coronary blood flow and oxygen demand, potentially triggering epicardial vasoconstriction. This combination of factors may also result in a reduced coronary arterial volume-to-myocardial mass ratio, altering the FFR measurement [46,47]. Poor spatial resolution caused by motion artifacts, such as those from respiratory or cardiac motion, can mimic focal stenoses and yield abnormal values [48]. Segmentation errors during modeling, especially in small branches, may exaggerate stenosis severity or miss functional lesions altogether [30]. These technical and patient-related factors highlight the necessity for optimal imaging protocols, careful patient selection, and further advancements in reconstruction and segmentation technologies to enhance the utility of CT-FFR in diverse clinical settings.

### 1.2. Significance of Plaque Burden

**Figure 3:**
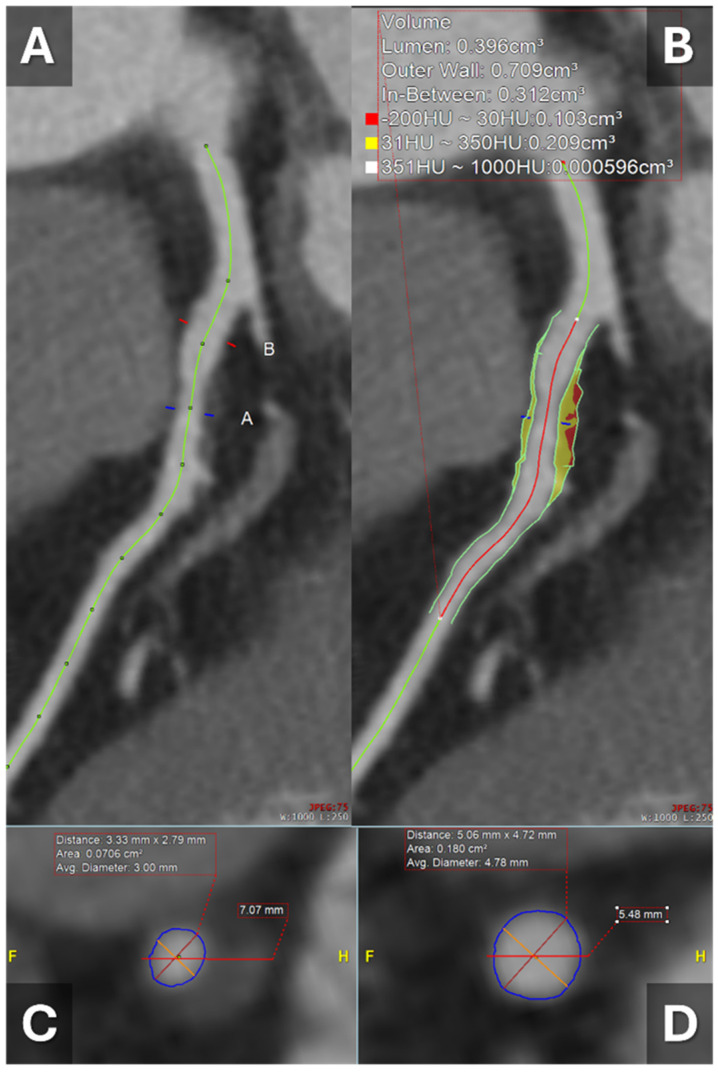
A 61-year-old male presented with atypical chest pain and proceeds to CCTA. He had moderate non-obstructive coronary atherosclerosis, which did not require revascularization; however, based on these findings, the patient was commenced on statin therapy and intensive risk factor modification. (**A**) Curve multiplanar reconstruction of the LAD demonstrating non-obstructive low-attenuation plaque at the level of “A” with luminal narrowing compared to level “B”. (**B**) Plaque analysis of the LAD stenosis showing low Hounsfield units, suggesting soft high-risk plaque with positive remodeling. (**C**) Cross-sectional imaging showing moderate stenosis at LAD level “A”. (**D**) Cross-sectional imaging showing no stenosis at LAD level “B”. **Abbreviations:** CCTA = coronary computed tomography angiography, LAD = left anterior descending artery.

The magnitude of plaque burden is a superior predictor of thrombotic events compared to stenosis grading alone [28]. Recent studies have highlighted that the total plaque burden, as assessed through coronary CT angiography, is strongly associated with adverse cardiovascular outcomes, including an increased risk of plaque rupture and myocardial infarction [28]. While severe luminal stenosis is often a late-stage manifestation of atherosclerosis, the extent and progression of plaque burden are more closely linked to future cardiovascular risk [28]. One study that evaluated 23,579 symptomatic patients who underwent CCTA revealed that calcified coronary plaque burden was amongst the primary predictors of future cardiovascular events [49]. Coronary artery calcium (CAC) scoring using CT offers the simplest method of assessing plaque burden. However, statin therapy may result in an increase in CAC that prevents its use as a method for longitudinal assessment of plaque burden. There is increasing evidence that plaque composition in addition to plaque burden plays a pivotal role in mediating the risk of adverse events, indicating that phenotyping of high-risk plaques that are unstable and vulnerable to rupture is critical in identifying those that are responsible for acute coronary syndromes and myocardial infarction (Figure 3) [50]. Furthermore, quantifying epicardial adipose tissue (EAT) via CT imaging has been associated with elevated risks of adverse cardiovascular outcomes, including a higher burden of coronary events and increased rates of revascularization [19,21,26,29,49]. Increased myocardial fatty acid uptake, linked to insulin resistance and impaired systemic fat storage capacity, contributes to cardiac dysfunction. Incorporating EAT quantification alongside traditional high-risk variables and coronary plaque characteristics may enhance the predictive accuracy for future cardiovascular events.

Despite remarkable advancements, significant challenges persist in the field of plaque imaging. Motion artifacts remain a major obstacle, particularly in patients with high heart rates or irregular rhythms. While innovations such as faster scanners and real-time motion correction algorithms have mitigated some of these issues, further refinements are necessary to achieve consistently high-quality images [43]. Radiation exposure is another concern, particularly for younger patients and those requiring frequent imaging. Techniques like PCCT and refined reconstruction have shown promise in lowering radiation doses without compromising image quality [43,51]. However, the implementation of these technologies on a broader scale is often limited by cost and resource constraints. The high expense of equipment and the need for specialized personnel further exacerbate the accessibility gap, particularly in resource-limited healthcare settings [43].

### 1.3. Artificial Intelligence and CT-Derived Plaque Analysis

Artificial intelligence (AI) has recently been investigated in regards to enhancing the detection and analysis of atherosclerotic plaques across various imaging modalities and improving diagnostic accuracy and workflow efficiency. The development of convolutional neural networks, a type of deep learning algorithm, has facilitated the handling of large imaging datasets and supported the creation of dedicated AI tools for medical imaging [52]. These advancements have improved plaque detection and analysis across various CT-based technologies, particularly CCTA. In CCTA, AI algorithms can aide in the detection and classification of coronary artery plaques, identifying high-risk features such as low-attenuation plaques and positive remodeling [52] (Table 2).

A recent meta-analysis assessed the diagnostic performance of artificial intelligence-assisted coronary computed tomography angiography (AI-assisted CCTA) for evaluating atherosclerotic plaque [55]. The analysis included 11 studies comprising 1484 patients and primarily focused on advanced AI methodologies, such as convolutional neural networks and other deep learning techniques, applied to CCTA images for detecting stenosis severity, calcification, plaque vulnerability, and characteristics, including calcified, non-calcified, and mixed components. The study reported a pooled sensitivity of 0.90 (95% CI 0.85–0.93), specificity of 0.93 (95% CI 0.87–0.96), and an area under the receiver operating characteristic curve (AUROC) of 0.96 (95% CI 0.94–0.97) for detection of high-risk features. Specifically, the AUROC for detecting stenosis of ≥50% was 0.95 (95% CI 0.93–0.96), for stenosis of ≥70% was 0.96 (95% CI 0.94–0.97), and for calcium detection was 0.92 (95% CI 0.90–0.94) [55].

In addition to plaque classification, AI-based methods have been shown to enable standardized quantification of coronary plaque burden, a predictor of cardiovascular outcomes [55]. An analysis of the DECODE study demonstrated that incorporating AI-based plaque quantification, specifically using the HeartFlow AI-QCPA (artificial intelligence–quantitative coronary plaque analysis) tool, into routine CCTA led to differences in medical management recommendations in 66% of cases [56]. This tool uses advanced machine learning algorithms to analyze CCTA images, with subsequent precise quantification of plaque characteristics and burden [56]. AI-based quantification methods have also shown excellent agreement with invasive IVUS, a gold standard in coronary imaging [56]. The REVEALPLAQUE2 and MIAMI3 studies validated the accuracy of HeartFlow’s AI-enabled quantitative coronary plaque analysis (AI-QCPA) in quantifying total plaque volume and classifying plaque subtypes, including calcified, non-calcified, and low-attenuation plaques [53,57]. Furthermore, AI reduces inter-observer variability, making plaque assessments more reproducible. Manual plaque measurements by CCTA readers are time-consuming and prone to variability, while AI offers a faster, automated, and more consistent approach to plaque evaluation [56].

Machine learning algorithms have advanced the calculation of CT-FFR without the need for time-consuming computational models. Machine learning-based CT-FFR allows for faster and more accurate calculations compared to invasive FFR and CFD-based methods, providing both anatomical and functional assessments [52]. AI-QCPA has further improved the clinical use of CT-FFR by offering additional insights into plaque characteristics that may not be apparent from stenosis severity alone. While AI-QCPA did not change CT-FFR ordering for stenoses ≥ 50%, it significantly altered decisions for stenoses < 50%, identifying high-risk plaques, such as non-calcified or low-attenuation plaques, that may require further evaluation [56].

AI technologies have also been investigated in quantifying CAC scores. Recent tools achieved an intra-class correlation coefficient of 0.98 when compared to expert readings and can be obtained accurately in a significantly reduced amount of time, improving clinical workflow [52,54]. Traditionally, CAC scoring is performed on ECG-triggered, non-contrast CT scans, requiring radiologists to manually identify high-attenuation areas in the coronary arteries. This method, while effective, is time-consuming and prone to variability between readers. AI-based automation addresses these limitations and may potentially improve the efficiency of cardiovascular screening programs [52,54].

## 2. Conclusions

Advancements in plaque imaging have significantly enhanced our ability to diagnose and manage atherosclerosis, shifting the focus from simply assessing luminal stenosis to understanding plaque composition and behavior. Emerging technologies such as spectral CT, PCCT, and hybrid imaging modalities provide key clinical data into vulnerable plaque characteristics, offering the potential for earlier detection and more precise risk stratification. Additionally, innovations like CT-derived fractional flow reserve and AI-driven analysis tools have further enhanced diagnostic workflows and improved the accuracy of clinical assessments. These efforts may guide individualized and effective care for coronary atherosclerotic care.

## Figures and Tables

**Table 1:** CT imaging modalities in plaque characterization.

Imaging Modality	Key Features	Clinical Utility	Diagnostic Performance	Limitations
Dual-Layer Spectral CT Angiography (DL-SCTA)	Differentiates calcified, non-calcified, and mixed plaques; identifies high-risk plaques using low attenuation values (<30 HU); assesses plaque burden and arterial remodeling.	Risk stratification, high-risk plaque identification, improved assessment of plaque burden.	Sensitivity 77%, Specificity 56% for high-risk plaques [4]; enhanced identification of lipid-rich plaques and neovascularization.	Lower specificity; limited data in certain populations; motion artifacts can impact image quality.
Photon-Counting CT (PCCT)	Measures individual photon interactions; improves spatial resolution and reduces noise; detects microcalcifications and thin-cap fibroatheromas with high sensitivity and specificity.	Early detection of vulnerable plaques, superior stent visualization, monitoring of disease progression.	Sensitivity 94%, Specificity 89% for lipid-rich plaques [5]; AUC 0.93 for CAD detection [6]; 100% sensitivity for stent patency [7].	Cost and availability; motion artifacts still a concern; limited widespread use.
Dual-Energy CT (DECT)	Utilizes X-rays at two distinct energy levels; enables material decomposition for precise plaque characterization; correlates plaque composition with myocardial infarction risk.	Identification of rupture-prone plaques, prediction of ischemic events, accurate tissue characterization.	Strong correlation with MI risk; RMSE < 5% for plaque component quantification [8]; ORs up to 20.0 for stroke prediction based on plaque features [9].	Radiation exposure; complexity in interpretation; limited by image noise and patient motion.
CT-Derived Fractional Flow Reserve (CT-FFR)	Combines anatomical and hemodynamic assessment; calculates functional significance of stenoses; improves specificity of CCTA and reduces unnecessary invasive angiography.	Improves clinical decision-making, guides revascularization, predicts major adverse cardiac events (MACE).	HR up to 5.05 for MACE with CT-FFR 0.80 [10]; improves specificity of CCTA; diagnostic accuracy reduced in high calcium scores or stents [11,12,13,14,15].	Accuracy impacted by high calcium or metallic stents; manual editing needed in complex cases; poor image quality reduces utility.

**Table 2:** Artificial intelligence-enhanced CT evaluation of plaque characteristics.

AI Application	Key Benefits	Study Findings
Plaque Detection and Classification	High sensitivity (90%) and specificity (93%) in detecting high-risk plaques.	Meta-analysis (1484 patients) reported AUROC of 0.96 for detecting high-risk plaques [53].
Quantification of Plaque Burden	Reduces inter-observer variability, aligns with IVUS standards.	AI-QCPA altered management in 66% of cases in DECODE study [54].
CT-FFR Calculation	Machine learning accelerates CT-FFR calculations, improving workflow efficiency.	AI-QCPA significantly changed decisions for <50% stenosis plaques [54].
Calcium Scoring	Automates and enhances calcium score accuracy, reducing reader variability.	AI achieved intra-class correlation coefficient of 0.98 with expert readings [52,54].
